# (Healthy) Aging Patterns in Europe: A Multistate Health Transition Approach

**DOI:** 10.1007/s12062-022-09403-4

**Published:** 2022-11-25

**Authors:** Aïda Solé-Auró, Jordi Gumà

**Affiliations:** DemoSoc Research Group, Department of Political and Social Sciences, Universitat Pompeu Fabra, C/ Ramon Trias Fargas, 25-27, 08005 Barcelona, Spain

**Keywords:** Health transitions, Aging patterns, Education, Gender, Panel data, Europe

## Abstract

We explore patterns and determinants of health transition probabilities by combining outcomes of morbidity and mortality to examine different aging patterns across Europe, and to ascertain how individual socio-demographic characteristics modify these patterns. We use panel data from the Survey of Health, Ageing and Retirement in Europe (2004– 2017) for 76,536 individuals aged 50 + in 20 European countries who participated in at least two waves. All transition probabilities were calculated applying a multistate analytical approach. Our findings show significant gender, education, and cohort differences in health transition probabilities and marked cross-country group differences. Central and Northern European countries present lower probabilities of health deterioration than Southern and, especially, Eastern European countries. Having a high level of education, living in Central Europe, and being younger are associated with lower probabilities of health deterioration and, if any, a higher probability of being restored to good health. We found less evidence of differences when transitions end in death. Our study contributes to this line of research by implementing a multistate approach using European harmonized panel dataset, to examine the effects of birth cohort, educational attainment and gender differences on health transitions. Our findings point to the need to consider the specific influence of individual factors in the aging process in different transitions according to the context and with reference to specific vulnerable groups. In the context of aging societies, such a consideration is both essential and policy relevant.

**Supplementary Information:**

The online version contains supplementary material available at 10.1007/s12062-022-09403-4.

## Introduction

As population health patterns worldwide have changed over the last few decades, studies of population trends seek to provide a better understanding of their dynamics. Population health profiles across Europe are highly heterogeneous, indicative of differences in demographic and epidemiological change across regions and, more than likely, of different aging patterns. Thus, Southern European countries, including Spain and Italy, enjoy high life expectancy, but present poorer population health profiles than their counterparts in Northern and Central Europe (Eikemo et al., 2008). Likewise, Eastern European countries present even worse health outcomes, but unlike Europe’s Southern region, the countries of Eastern Europe also have the worst mortality rates on the continent (Mackenbach, 2017; Permanyer et al., 2018).

In this context, the study of health transitions and their determinants is central to policy debate in most of these economically advanced regions. Indeed, it is expected that certain groups of individuals (or countries) that experience a later transition to poor health present a better health profile or, at least, a greater compression of morbidity to end of life. Given these forecasts, the identification of modifiable factors that can reduce or delay the transition from good to poor health or from poor (or good) health to death is clearly critical for improving the current and future quality of life in our societies. Since the aging process coexists over the life-course with specific and time-varying individual and contextual characteristics (Alcañiz & Solé-Auró, 2018), scholars stress the need to monitor the socio-demographic sources of individual (socioeconomic and gender) and contextual (country) health inequalities. Moreover, studies to date have focused mainly on transitions related to deteriorations in health; yet, other types of transition, such as recovery (Doblhammer et al., 2009), or differences across educational groups, have received less attention.

This paper contributes to the existing literature by addressing the following two research questions. First*,* taking a longitudinal perspective and using multistate Markov models, we calculate health transition probabilities in order to explore how different European aging patterns vary. In so doing, we consider both deteriorations in health and recoveries (the focus of far fewer studies), and the final transition to death. Second, we examine how the cohort of birth and the level of education that act as sources of social and health inequalities modify or shape these aging patterns in four groups of European countries. The specific factors considered are birth cohort, educational attainment, and gender differences by region. Understanding the mechanisms associated with these health transitions could help reduce health inequalities between groups and delay the onset of the negative effects of aging on health, thus permitting an improvement in society’s well-being thanks to an overall improvement in the population’s health.

## Background

The study of individual characteristics – gender, age, education, and geographical location – is important for understanding their role as drivers of health transitions. Below, we address the theoretical links between these four socio-demographic factors and health and we identify notable gaps in current knowledge.

### Health Transitions

Understanding how health transitions change over time is crucial for assessing the future social and healthcare demands of Europeans. This study examines four health transitions (health deterioration, health recovery, and absorbing state) between three health states (being in good health, being in poor health, and death). This contributes to the existing literature when examining the effects of birth cohort, educational attainment, and gender on health transitions. The conceptualization of a health transition might differ from one socio-demographic group to another when defined by these factors. For instance, education is associated with changes in health, since the risk of transitioning to death from either health status (good or poor) is higher among those with low levels of education and increases with age (Doblhammer et al., 2009). Thus, we hypothesize that improvements in health are more likely among those with high levels of education and among later-born cohorts. Additionally, the study of health transitions seeks to account for the gender health survival paradox (Oksuzyan et al., 2008). Consequently, we hypothesize a greater magnitude in health deterioration among women, given that, in general, they are in worse health, and higher probabilities of health recovery among men but, paradoxically, also, of mortality. However, these settings might vary from one society to another depending on their stage of social development and aging process. The study of health transitions seeks to confirm the existence of three aging patterns across countries: low degree of health deterioration and low mortality (Northern and Central European countries); high degree of health deterioration but low mortality (Southern European countries); and high degree of health deterioration and high mortality (Eastern European countries). With this information, policymakers should be better able to adapt their social and health interventions, reduce health inequalities between different socio-demographic groups and achieve health equity.

Health, a multi-dimensional concept that defines a state of complete physical, mental and social well-being (McCartney et al., 2019; Ware, 1987), is complex, which makes understanding cross-national variations challenging because they are driven by multiple determinants (such as, education) that vary across and within national populations. Given this complexity, this study uses self-perceived health as it easily captures the general state of health during the disablement process (Jagger et al., 2011). Self-perceived health, moreover, has been reported to be a good indicator of quality of life at advanced ages and to be an insightful approach to measuring population health (Feng et al., 2016; Wu et al., 2013; Yamada et al., 2012). There are two primary reasons why the study of health transitions based on this particular health outcome are of interest to us: first, self-perceived health has already been shown to be a solid predictor of mortality (Jylhä, 2009; DeSalvo et al., 2006); and, second, in recent decades, increments in poor self-assessed health and activity limitations over time among adult and older people have been found (Mackenbach et al., 2018; OECD, 2019), highlighting the need to understand the deterioration in health during the disablement process (Verbrugge & Jette, 1994).

### Association between Education and Gender in Health Across Regions

Education has been identified as one of the strongest predictors of health and mortality as well as an important predictor of adult experiences, since it both mediates and moderates the health consequences of early-life disadvantages (Montez & Hayward, 2014). However, although it is one of the most frequently studied social determinants of health, educational attainment appears to have a distinct influence on health transitions depending on the specific health outcome under focus. For example, older individuals with a lower level of education generally report to be less likely to recover from a pre-frailty status (Ikeda et al., 2019); while individuals with a higher education status present a delayed progression to mild cognitive impairment and a lower risk of transitioning from normal to mild impairment (Robitaille et al., 2018).

Previous studies of the links between health and education identify the latter as a key determinant of health inequalities within European regions. Indeed, education expanded greatly in the twentieth century, especially among women, albeit the process adhered to a different timeline across the continent (being recorded first in the countries of Northern and Central Europe) (Meschi & Scervini, 2014). The regions where this expansion was initiated earliest present lower health inequalities by education level (specifically, countries with Bismarckian welfare regimes), while regions where the expansion of education was delayed (i.e. the Southern European countries) present greater health inequalities by education level (Eikemo et al., 2008). However, an overall pattern in Europe over the last century shows that the expansion of education reduces any direct association between education and health across cohorts and leads to a reassessment of the beneficial effects of education on health (Delaruelle et al., 2015). Focusing on health transitions in different populations, education might have a differential impact on the magnitude of these inequalities in the countries of Northern, Central, and Southern Europe, presenting a significant influence only in these last two groups of countries (Avendano et al., 2009). Indeed, Central and Southern Europeans aged over 50 with low levels of education present higher probabilities of experiencing incident events of poor health, chronic disease, and disability, while their Nordic counterparts do not present these same health disadvantages. This difference can be explained by a weaker association between poor levels of education and financial and employment disadvantages in Northern countries compared to Central and Southern Europe. In line with resource substitution theory (Ross & Mirowsky, 2010; Ross et al., 2012), education also has gendered effects on health. For instance, lower female participation in the labor market, in addition to the unequal gender wage gap, has reinforced the importance of education for women’s health.

Gender is another factor that contributes to understanding health inequalities (transitions) across and within populations. Being male or female can modify the path of the health transition and also be a determinant of when a transition occurs. This is not only because women live longer than men, but also because women face worse health conditions (Oksuzyan et al., 2010; Oksuzyan et al., 2018), a situation that is dependent on the complex interrelationship between the social, behavioral, biological and epidemiological context of their geographic location (Crimmins et al., 2011, 2019). For instance, studies generally report significant gender differences in self-perceived health in most European countries; however, Bambra and Eikemo (2009) found no gender differences in this specific health outcome in the cases of Belgium, France, and Germany at younger adult ages. A similar finding was reported by Adjei et al. (2017) at ages 65 and over in the cases of Italy, Spain, and the UK.

Thus, as suggested in the preceding discussion, there are potential interactions between the proposed determinants. Education is widely believed to play a crucial role in defining social stratification and health inequalities; yet, the links between education and the welfare state are complex. Indeed, the institutional design of training systems and education has implications for social inequality that can impact policy making. The expansion of access to higher education is not necessarily the panacea for inequality, because the redistributive implications of investment in higher education are complex and not equally distributed across countries. At the same time, while the expansion of education has been greater among women, this process has occurred more rapidly than the decline in the levels of general gender inequality across Europe. All in all, this points to a highly complex scenario that is also subject to change over time.

Due to the lack of longitudinal incidence data on health in Europe, evidence of health transitions across cohorts is scarce. The little research evidence there points to a general health improvement among later-born cohorts compared to that of earlier cohorts (Chatterji et al., 2015). In one of the few studies undertaken, Ahrenfeldt et al. (2018) compared two birth cohorts using two waves of the Survey of Health, Ageing and Retirement in Europe (SHARE) (2004 and 2013) for individuals aged 50-plus. The authors reported improvements in cognitive functioning among later-born cohorts, but a stable or unclear trend in limitations affecting activities of daily living (except among the oldest-old living in Nordic countries). However, the authors stress that their study does not consider the health variation within the wide range of age groups (50–90) in their data; hence, their results could be influenced by the age effect and not necessarily show a pure cohort effect.

Age constitutes another key factor when analyzing health longitudinally. For instance, while the risk of dying increases exponentially with age (Ledberg, 2020), when other socioeconomic factors (e.g. education) are included the age effect becomes less evident. Additionally, age has been identified as a possible leveler of the influence that other factors exert on mortality and health deterioration (the higher the age, the lower the capacity of social factors to define self-perceived health differences) (Lynch, 2003). However, recent research has shown how factors, such as education, reduce the effect on health differences due to a cumulative effect of health disadvantages over the life course and a consequent survival selection at old ages (Dupre, 2007; Ferraro & Farmer, 1996).

## Methods

### Data

We use harmonized data taken from SHARE, a longitudinal survey representative of the non-institutionalized population aged 50 and over (Börsch-Supan & Jürges, 2005; Börsch-Supan et al., 2005). We combine data from the first (2004) to the seventh (2017) waves of SHARE to illustrate differences in these novel, comparable measurements of gender inequality in health. We did not consider data from the eighth wave as it was interrupted by the COVID-19 pandemic. Note that while there was a two-year gap between each of the waves, the second was an exception, being conducted some three years after the first. In order to analyze possible health transitions over time, we pooled the information for all individuals that were interviewed at least twice.

SHARE began collecting data in 2004 (wave one) in 12 European countries. In subsequent waves, SHARE incorporated other countries – among these, the only one not in Europe is Israel, which was accordingly discarded from our analysis. Note, however, that countries whose participation began in the seventh wave are likewise not included here, as these respondents have only been observed on one occasion. Moreover, the 20 countries eventually included in our analysis were selected because of their relatively high response rate in the SHARE baseline interview. In our analysis, we refer to these 20 countries collectively as “Europe.”

We use data for men aged 50 to 79 and for women aged 50 to 84. We opted for different cut-off ages because of different average life expectancies at birth. By not extending our analysis beyond these age boundaries, we avoid underestimating the mortality transition, especially for the oldest age group. The final sample is composed of 76,536 individuals (54.8% women), while the country sample sizes with a unique person-observation over time range between 698 for Ireland and 6,937 for Spain. In our analysis, we omitted some individuals because they had been interviewed only once (the main source of missing data), their data were incomplete, or because they lay outside the age range (32 and 30% of female and male respondents, respectively).

The SHARE survey suffers from a reduction in the number of individuals followed up over time due to attrition. This level of attrition tends to be highest among first-time participants and falls as individuals participate in more waves (Bergmann et al., 2019). However, attrition has been shown to have little, if any, selection bias effect on the overall sample composition (Kneip et al., 2015), which confirms the adequacy of SHARE data for our purposes here, particularly for men younger than 80 and women younger than 85. As a robustness check, ideally, we would have weighted for the whole population; however, SHARE only provides longitudinal weights for consecutive waves, so we are unable to apply weights for respondents participating in non-consecutive waves. There are two potential reasons for this: first, the intermittent participation of individuals/countries in SHARE (e.g. a person or country that participated in wave 1, but not in wave 2, yet returned to participate in wave 3); and, second, individuals that died in the period between waves, who are of particular interest in our analysis. This represents about 15% of all person-observations in our sample and, because of this high proportion, weights were not used in our analysis.

### Measures

Our analysis uses two harmonized measures over time. First, we use self-perceived health, where respondents provide an overall subjective assessment of their health in answer to the question: “Would you say that your health is excellent, very good, good, fair or poor?”. Responses were grouped into two categories: good (excellent, very good or good) and poor health (fair or poor). This measure provides a good summary and integrates a global health perspective (De Bruin et al., 1996), unlike other measures, such as chronic conditions and mobility limitations, that provide a complementary vision of health. Second, we use information about the death of sampled individuals to define all possible health transitions.

To obtain a more complete view of the overall disablement process, we performed a robustness check by adding two additional objective health measures of problems with functioning and disability. First, difficulties in performing at least one of six activities of daily living (ADLs), indicative of an individual’s ability to care for themselves. The ADL functions include walking across a room, getting in and out of bed, bathing or showering, eating (including cutting up food), dressing (including putting on shoes and socks), and using the toilet (including getting up or down). Second, the Global Activity Limitation Indicator (GALI), which measures health-related activity limitation with a single question: ‘For at least the past 6 months, to what extent have you been limited because of a health problem in activities people usually do?’ (severely limited; limited but not severely vs not limited). Tables S1 to S4 of the supplementary materials provide the results of our multistate models for the Global Activity Limitation Indicator (GALI) and limitations in Activities of Daily Living (ADLs), respectively. Overall, the resulting transition probabilities confirm that the level of education achieved, the birth cohort and country group gradient observed with self-perceived health follow a similar pattern when focusing on these two additional health measures.

### Variables of Interest

We examine the role played by four socio-demographic variables as drivers of health transitions: the individual characteristics of gender, level of education achieved, and birth cohort, plus geographical location (country group) to account for an individual’s common circumstances over their life-course.

Education is our measure of social class. Formal education is acquired relatively early in the life course but is suitable as an indicator of socioeconomic status even in older age. We consider three groups defined in accordance with the International Standard Classification of Education (ISCED)^1^: *low* (corresponding to ISCED 0–1, no or primary education, and ISCED 2, lower secondary education), *medium* (ISCED 3–4, higher secondary education), and *high* (ISCED 5–6, tertiary education – reference). We opted not to use other variables, such as income or occupational status, as there are substantial changes across birth cohorts and, particularly, between men and women (for instance, the labor force participation of women was very low in the first birth cohort considered).

We also include three birth cohorts to account for both age and the individual life-course circumstances in different regions. By so doing, regardless of when the respondents joined the SHARE sample, or the year when each country began to participate in SHARE, individuals are grouped within the same date-of-birth ranges. Thus, we avoid including in the same age group individuals who report being the same age in different years of observation (e.g. 2004 and 2017) and in different waves of the survey. Hence, the older the birth cohort, the older the individual. We include the birth cohort to account for the different historical circumstances in which the life trajectory of respondents has been played out; but, as we conduct a longitudinal analysis, age is also implicit to our study. In the analysis reported here, we employed three birth groups of similar sample size: 1920–1939 (33% of the total sample born before World War II); 1940–1949 (32.5% of the total sample born during and immediately after World War II); and 1950–1962 (34.5% of the total sample born in the second half of the last century). It should be stressed, however, that the time interval of the survey (2004 and 2017) considered here does not permit us to compare cohorts, as we are unable to observe the respondents in the three groups over the same age ranges.

The 20 European countries included in our analysis are classified into four groups on geopolitical grounds and by type of welfare state regime (Esping-Andersen, 1990; Schmitz & Lazarevič, 2020): Northern European (Denmark and Sweden); Central European (Austria, Belgium, France, Germany, Ireland, Luxembourg, the Netherlands, and Switzerland); Southern European (Greece, Italy, Portugal, and Spain); and Eastern European (Croatia, Czech Republic, Estonia, Hungary, Poland, and Slovenia).

### Analytical Strategy

In our analysis, we first describe the characteristics of our working sample, focusing primarily on percentages of poor health in each wave of SHARE and the number of deaths between waves. This focus is essential for determining transition probabilities, since they are not independent of the baseline distributions. Second, we compute the health transitions of European men and women, which we class in one of four categories according to the change in health over time at the population level (Crimmins et al., 2010).

**Fig. 1:**
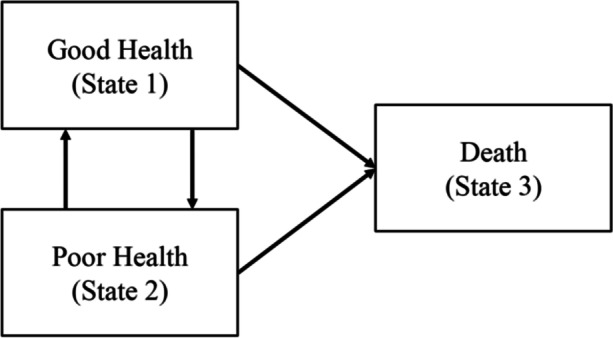
Dimensions of the health transition states. Source: Authors’ own elaboration

Figure 1 shows the possible transitions between the three health states – being in good health, being in poor health and death – that an individual can experience. Death is an absorbing state (that is, no other transition is possible once an individual enters this state). Individuals can remain in the same state without moving (i.e. remain healthy or unhealthy), but in case of change, our analysis focuses on four possible transitions: *health deterioration*: when transitioning from good to poor health; *health recovery*: when transitioning from poor to good health; and *absorbing state*: when transitioning from either good or poor health to death (Fig. 1).

We then proceed to calculate the probabilities of these health states for all possible transitions according to our variables of interest (age and level of education) for both women (aged 50–84) and men (aged 50–79) in each country group. All transition probabilities were calculated using multistate Markov models for panel data where both models (women and men) included the three covariates. In general, multistate model likelihood is calculated from the transition probability matrix P between two points in time, t_1_ and t_2_, where t_2_ is equal to t_1_ plus the time interval between two panel data. For each individual, the (h, i) entry in the probability matrix P of a multistate model is the probability of their declaring to be in state i at t_2_, considering that their previous state at time t_1_ was h.

$${\mathrm{p}}_{\mathrm{ih}}({\mathrm{t}}_{1}, {\mathrm{t}}_{2})=\mathrm{P}[{\mathrm{X}}_{\mathrm{t}2}=\mathrm{h }| {\mathrm{X}}_{\mathrm{t}1}=\mathrm{i}]$$

P(t_1_, t_2_) is calculated in terms of the matrix of transition intensities (Q) using the Kolmogorov differential equations (Cox & Miller, 2017), where the intensity represents the risk of moving from state h to i when h ≠ i. These intensities are calculated as follows:

$${q}_{ih}\left(t,z\right)=\underset{\mathit{\delta t}\to 0}{\mathrm{lim}}P\left(S\left(t+\delta t\right)=h \right| S\left(t\right)=i)/\delta t,$$

where z is an explanatory variable (here, either education, gender, birth cohort or country group).

Finally, if the transition intensity matrix Q is constant over the interval (t_i_; t_j_), as in a homogeneous process, then P(t_i_, t_j_) = P(t) and the equations are solved by the matrix exponential of Q scaled by the time interval:

$$\mathrm{P}(\mathrm{t})=\mathrm{Exp}(\mathrm{tQ}).$$

The likelihood, then, is the product of transition probabilities between all possible observed states, for all individuals (i) and observation times (j):

$$L\left(Q\right)=\prod_{i}{L}_{i}=\prod_{i,j}{L}_{i,j}=\prod_{i,j}{ps(t}_{ij}){s(t}_{i,j+1}){(t}_{i,j+1}-{t}_{ij})$$

where each component L_i,j_ is the entry of the transition matrix P(t) at the S(tij)th row and S(t_i,j+1_)th column, evaluated at t = t_i,j+1_ – t_ij_.

Where the specificity of the panel data is that the different possible states for an individual are only known at a finite series of times t = (t_1_;…; t_n_), instead of being continuously observed over a fixed period of time. In this case, fitting a multistate model to panel data relies on the Markov assumption that future evolution only depends on the current state, which simplifies the process as the probabilities obtained are independent of the previous observation history of the process up to time t.

Thus, the advantages of using multistate Markov models for panel data in our research are threefold: first, it allows us to account for the fact that, here, the only transition for which we know the exact time of occurrence is death, whereas the transitions from one state of health to another are compiled at the time of the survey and not when they actually occurred; second, it also allows us to undertake a combined analysis of data from all SHARE countries regardless of the wave in which their participation began or the continuity of their participation (e.g. Greece, Poland, the Netherlands and Portugal’s participation in SHARE was discontinuous over the time period analyzed); and third, it allows us to analyze individuals with different observation patterns (i.e. a different number of participations in SHARE over time, differences in time gaps between observations for individuals with a continuous or discontinuous participation in the survey, etc.). Thus, the only restriction we impose is that respondents in SHARE countries had to have been interviewed at least twice so that we could determine any possible transitions (Jackson, 2011).

We initially verified that our variables of interest presented significant differences in the hazard ratios between transition probabilities by calculating specific multistate models for cohort of birth, education level and country group separately by gender (results available upon request). In general, we detect statistically stronger differences for transitions between different health statuses than when the final state is death. This also appears to be true when we consider the other two health measures, ADL and GALI (results available upon request). All statistical analyses were performed using the MSM package in R version 1.6.7.

## Results

### Descriptive Findings

Table 1 shows the characteristics of our working sample (with prevalence expressed as percentages together with the average number of individual participants) according to level of education, birth cohort and country group for men and women. The overall distribution in education presents a number of differences by gender, with more women than men in the lowest education category (28.8 vs. 23.4%, respectively). However, roughly 20% of all respondents (18.1 women vs. 22.9% men) are assigned to the highest level, while those with a medium level of education account for the majority of the sample for both genders. As for the countries assigned to four geographic groups, the largest in terms of both the number of countries and individuals corresponds to the Central European grouping. Although the number of countries in the Eastern European group doubles that of the Southern group, the two present similar absolute and relative numbers of participants. This is attributable to the fact that Spain and Italy have participated in SHARE since its first wave. The Northern group comprises just two countries, but again, as they have participated in SHARE since the first wave, the number of observations per individual counterbalances this. Finally, on average, women participated 3.8 and men 3.7 times in our working sample across the seven waves.

**Table 1:** Sample characteristics by education level, cohort of birth and country group for men and women. Individuals aged 50–84

		Women		Men	
		Absolute	Relative (%)	Absolute	Relative (%)
Education Level	Low	12,082	28.8	8,101	23.4
	Medium	22,272	53.1	18,581	53.7
	High	7,588	18.1	7,928	22.9
	Total	41,942	100	34,610	100
Cohort of birth	1920–39	13,973	33.3	11,287	32.6
	1940–49	13,271	31.6	11,603	33.5
	1950–62	14,698	35.1	11,720	33.9
	Total	41,942	100	34,605	100
Country group	Central	17,415	41.5	14,661	42.3
	Northern	5,113	12.2	4,521	13.1
	Southern	9,174	21.9	7,919	22.9
	Eastern	10,240	24.4	7,509	21.7
	Total	41,942	100	34,610	100
Average nº of participations in SHARE sample		3.76		3.69	

Source: Pooled data from the first to the seventh waves of SHARE (2004–2017)

Table 2 reports the prevalence of poor self-perceived health by education, birth cohort and country group for each of the seven waves of the SHARE survey by gender. Results confirm an educational and birth-cohort gradient for all seven waves analyzed, with individuals with a higher education and those born in younger cohorts showing a lower prevalence of poor health. For both genders, Northern European countries present the lowest values of poor self-perceived health, while the Southern and, in particular, the Eastern European countries present the highest. In fact, none of the Eastern European countries was sampled in the first wave, but their progressive incorporation coincides with a rise in the overall prevalence of poor self-perceived health. Finally, while all these patterns are common to women and men, the former report higher percentages of poor self-perceived health than males, while the gender gap is widest in the most disadvantaged categories (low education and 1920–39 birth cohort). Finally, the Southern European countries show the greatest gender difference when we consider the country group values.

**Table 2:** Prevalence of poor self-perceived health (%) in each SHARE wave by education level, cohort of birth and country group for men and women. Individuals aged 50–84

			Wave 1	Wave 2	Wave 3	Wave 4	Wave 5	Wave 6	Wave 7
Women	Education	Low	42.2	49.3	55.1	55.7	54.9	56.1	55.4
		Medium	24.1	31.2	37.1	39.3	37.9	40.6	41.0
		High	13.4	17.9	24.4	25.8	23.8	25.3	28.4
	Cohort	1920–39	37.5	47.3	53.1	55.5	55.8	59.6	60.3
		1940–49	24.9	31.9	37.2	38.8	37.8	41.0	41.6
		1950–62	18.9	24.3	30.4	31.1	28.3	30.3	31.3
	Country group	Central	26.3	30.2	35.8	30.7	31.5	32.1	34.7
		Northern	15.1	26.1	32.8	26.9	24.0	26.2	29.0
		Southern	40.5	42.8	47.2	50.9	49.0	48.1	49.5
		Eastern	-	49.6	54.6	55.7	53.8	57.2	52.5
	Total		**28.6**	**35.3**	**40.8**	**41.0**	**38.9**	**41.6**	**42.0**
Men	Education	Low	32.3	40.7	47.0	47.9	45.6	46.4	49.9
		Medium	20.5	27.1	33.6	36.6	35.4	37.5	38.6
		High	12.4	16.3	22.9	22.1	21.6	24.6	28.3
	Cohort	1920–39	27.5	37.1	43.7	45.2	44.9	48.0	51.4
		1940–49	20.2	26.5	32.5	34.4	33.8	37.1	38.5
		1950–62	15.3	20.4	26.7	30.1	27.8	29.2	31.8
	Country group	Central	23.0	26.0	32.1	27.4	28.1	29.6	33.2
		Northern	10.7	19.9	25.8	21.8	20.1	21.0	25.9
		Southern	26.3	29.5	35.1	39.4	38.3	37.5	40.9
		Eastern	-	46.4	52.7	52.7	52.0	55.7	51.6
	Total		**21.6**	**28.2**	**34.3**	**35.4**	**33.7**	**36.1**	**38.0**

Source: SHARE data (2004–2017)

### Transition Probability Outcomes

**Fig. 2:**
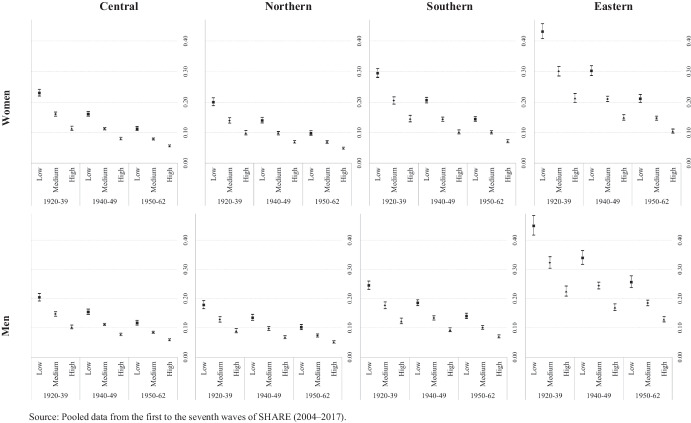
Transition probabilities from good to poor health by education, cohort of birth and country groups. Source: Pooled data from the first to the seventh waves of SHARE (2004–2017)

**Fig. 3:**
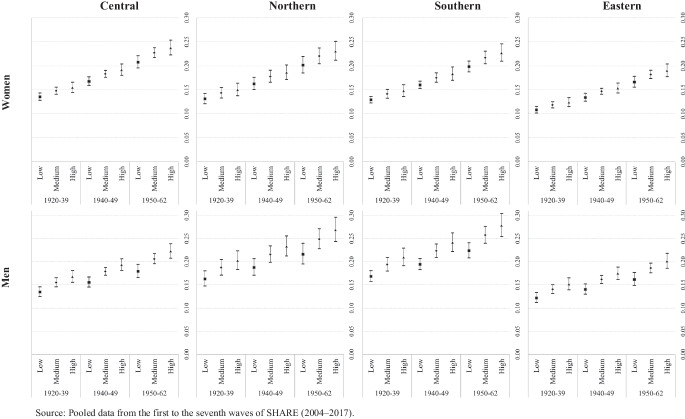
Transition probabilities from poor to good health by education, cohort of birth and country groups. Source: Pooled data from the first to the seventh waves of SHARE (2004–2017)

**Fig. 4:**
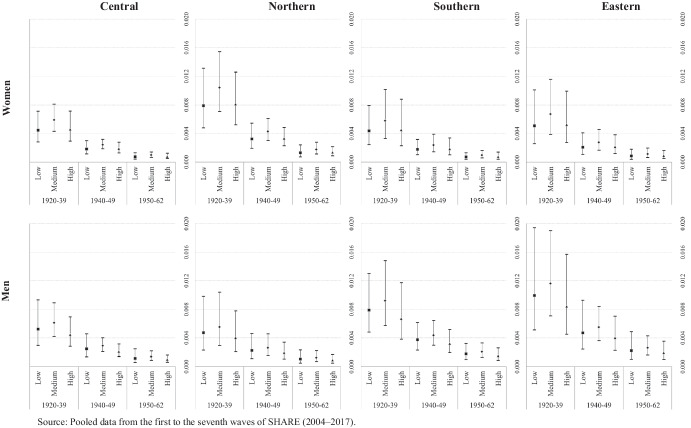
Transition probabilities from good health to death by education, cohort of birth and country groups. Source: Pooled data from the first to the seventh waves of SHARE (2004–2017)

**Fig. 5:**
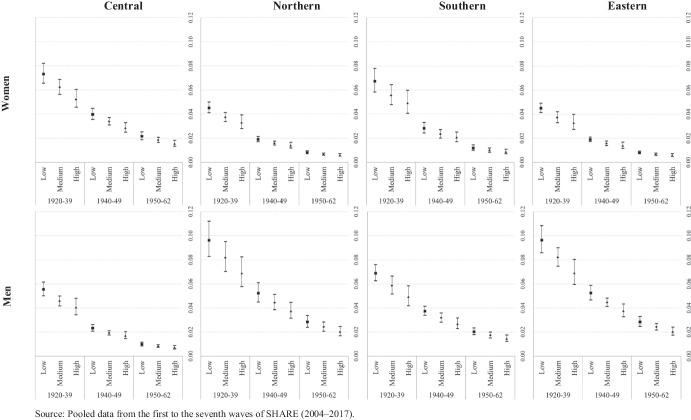
Transition probabilities from poor health to death by education, cohort of birth and country groups. Source: Pooled data from the first to the seventh waves of SHARE (2004–2017)

Figures 2, 3, 4, 5 show the transition probabilities analyzed herein for each state (i.e., deterioration, recovery, and absorbing state) (Tables S5 and S6 present these probabilities and their confidence intervals for women and men, respectively). Although education, cohort of birth, and country group are all covariates in our models, the results are grouped by European region to facilitate their visualization. Figure 2 shows the predicted transition probabilities from good to poor health for men and women according to their country group by educational attainment and cohort of birth. In general, we detect a clear gradient in terms of the magnitude of the probability of deterioration by cohort of birth and education. If we focus on birth cohort, those in the older cohort invariably present higher probabilities of reporting a deterioration in health, which is indicative of a clear age effect. In the case of educational attainment, the trends within each cohort of birth present significant differences in all four country groups. However, a general pattern emerges, albeit with different magnitudes, whereby the higher the level of education the better the health profile (defined by a lower probability of identifying a transition to poor health). We should stress that our findings with regard to education, particularly high levels of attainment, seem to counter the detrimental effect of having born earlier. For instance, in all country groups and for both genders, individuals with high levels of education in the first birth cohort group (1920–1939) present similar probabilities of declaring a health deterioration to those with a medium education in the second group (1940–1949) and to those with a low level of education in the third group (1950–1962).

Figure 2 also shows substantial differences across country groups, with Central and Northern European countries presenting lower probabilities of health deterioration than those presented by countries in the South and, especially, those in the East of Europe. Indeed, while all four country groups present the same probability gradients according to education and age, the magnitude of this gradient is considerably more pronounced for Eastern Europeans of both genders.

Finally, Fig. 2 also illustrates the gender patterns associated with the magnitude of the probability of worsening health in the four country groups. Men and women present similar transition probabilities to poor health in Central and Northern Europe. Southern European women with low or medium levels of education present higher significant values in the two older cohorts than those presented by their male counterparts; however, in Eastern Europe, it is the women that present lower probabilities of deteriorating health, most notably in the two youngest birth cohort groups.

Figure 3 shows the predicted transition probabilities from poor to good health for men and women according to their country group by educational attainment and age. Here, regardless of gender and country group, we see a clear, positive linear gradient for the probability of health recovery by birth cohort and education. This indicates that in all cases the younger the cohort, the greater the probability of transitioning from poor to good health, while within each cohort, those with a high level of education have a higher probability of being restored to good health. Despite this general pattern, differences in the magnitude of the probability of health recovery can be detected according to gender and country group. For example, Southern European countries present the largest gender gap (with men showing a higher probability of recovery than women) regardless of birth cohort and level of education. We detect higher probabilities for Northern and Eastern European males in the oldest cohort, while Central European countries only display a significant gender difference in the case of individuals with low or medium levels of education in the youngest cohort. Finally, country differences in magnitude are also present particularly among men in Eastern Europe, who report lower probabilities than those reported by individuals in the other country groups for all combinations of birth cohort and level of education.

Figures 4 and 5 show the results for health transitions to the absorbing state, that is, transitioning from either good or poor health to death, respectively. When individuals initially reported themselves to be in good health, it seems that their predicted probabilities of death are defined mainly by their cohort of birth rather than by their level of education. This applies to both genders and all four country groups (Fig. 4). However, the absence of differences in transitioning to the absorbing state, especially among individuals in the oldest cohort, could be due to the small number of individuals who actually died transitioning from good health – this expands the confidence intervals for the predicted probabilities. When transitioning to the absorbing state from poor health, combining the three birth cohorts and the three levels of educational attainment results in a linear gradient of predicted probabilities for both gender and country group (Fig. 5). This indicates that the lower the level of education and the older the individual, the greater the probability of transitioning to death. Moreover, this health transition presents the clearest gender gap, with men presenting the highest probabilities of dying when they declare themselves to be in poor health in all country groups and for all education levels and cohort of births groups.

## Discussion

This paper addresses health inequalities by analyzing patterns and determinants of health transitions among older Europeans. Our findings suggest significant gender, education and age differences in the effects (and magnitude) of the four health transition probabilities considered. In line with previous findings on health outcomes (Mackenbach, 2017), we additionally detect marked differences across country groups, with the countries of Central and Northern Europe presenting lower probabilities of health deterioration than those presented by their counterparts in the South and, above all, the East of the continent. Having a high level of education, living in Central Europe and being younger are associated with lower probabilities of health deterioration and – when reported – with higher probabilities of being restored to good health. However, we observe less evidence of differences, beyond those associated with age, when transitions end in death.

While some of the cohort and educational attainment effects found in our study were expected a priori, we should, nevertheless, highlight the country group differences observed. The fact that Southern European countries present higher probabilities of health deterioration than those presented by their Central European counterparts seems to be indicative of different patterns of aging (and health diseases) across European countries. For instance, we observe and reaffirm that Central European countries present the best outcomes in terms of health (i.e., low probabilities of deterioration and high recovery rates) but record higher mortality levels than those of Southern Europe. Paradoxically, Southern Europeans present low mortality rates but a worse path in terms of health (Crimmins et al., 2011; Mackenbach, 2017). Finally, Eastern European countries show themselves to be at a marked disadvantage in terms of both mortality and health status.

More specifically, our findings show the health penalty of the transition from good to poor health to be more apparent for the oldest cohort (born 1920–1939), with the education gap again being evident in Southern and Eastern Europe, the two country groups in which the expansion of education was most delayed. Here, education might counterbalance the age effect, with older cohorts showing similar probabilities of health deterioration to those in the immediately younger group with a lower level of education.

Health recovery rates show similar cohort and education patterns in the four country groups analyzed, although the magnitude of this transition is significantly lower in the countries of Eastern Europe, where the effect of education on these transitions appears to weaken. Thus, in terms of the transitions between the two health statuses other than death, education seems to be primarily a factor of prevention rather than recovery, while for those living in one of the Eastern European countries it is the factor that makes the difference. The higher mortality rates in this country group might be attributed to the double burden resulting from the lower rate of health recovery and the higher probability of heath deterioration, although this needs to be confirmed in future studies.

Likewise, the influence of education on health transitions that end in death is weak or almost non-existent. The health penalty of the transition from good health to death appears to be dependent mainly on age (i.e. the earlier the birth cohort, the higher this probability becomes). The small educational gradient found in the transition from good health to death is interesting and might be attributed to the fact that these individuals die from more sudden, or less preventable, causes (Mackenbach et al., 2015). Finally, as expected, the transition from bad health to death appears to be highly dependent on birth cohort, with higher values for the countries of Eastern Europe, in particular, and those of Northern Europe. However, the lower probability of health deterioration recorded by the Nordic countries seems to be counterbalanced by low mortality levels. Our findings are in line with previous research that shows that it is health, and not socio-economic factors, that accounts for the mortality differences between individuals at old ages (Rehnberg, 2020).

In sum, our findings suggest that education postpones the deterioration in health (from good to poor health) associated with age but that it has no positive effect once individuals are already in poor health. The health advantage enjoyed by individuals with high levels of education or by those living in Northern and Central Europe is attributable to both a lower probability of health deterioration and a higher probability of health recovery than those of their counterparts in Eastern European countries. Yet, in general, in the transition to death, these two factors do not seem to make any difference, which, in line with expectations, is often the case when the transition to death is from a state of poor health.

We detect similar magnitudes of transition probability between the two health statuses for men and women with the positive and significant effects of educational attainment and country group. This indicates that mortality and, specifically, the transition from poor health to death is the main mechanism accounting for the male–female survival-mortality paradox: while health deterioration and recovery rates are similar for both genders, the fact that older women spend more time in poor health is attributable to their later death once they experience a deterioration in health.

Our focus on educational attainment, gender, and country groups as markers of adult circumstances reflects several considerations outlined in the introduction. Our results strengthen the view that the study of health transitions constitutes a promising strategy for shedding new light on the understanding of the individual’s life course and population health outcomes in the West (Johansson et al. 2003). In general, delaying the deterioration in health by using education as a driver seems to be a good mechanism and one that allows us to understand the education gradient in health and mortality and the associated country differences (Mackenbach, 2017; Solé-Auró et al., 2015).

In addition, the marked effect of education on the probabilities of recovering from poor self-perceived health in Southern Europe, the country group with the fewest individuals with high or medium levels of education in our working sample, confirms the differential magnitude of this social determinant of health, which is dependent on the socioeconomic context (Avendano et al., 2009). The high levels of socioeconomic inequality in Southern Europe might, in part, account for this result, as previously suggested when analyzing socioeconomic differences in quality of life using SHARE data (Niedzwiedz et al. 2014). However, this is not confirmed for women, although the high share of traditional gender norms in a Southern European country, such as Spain, has been shown to emphasize the importance of a partner’s education for understanding health differences among old women (Gumà & Spijker, 2019). Thus, high levels of contextual gender inequality in some regions could mask the real effect of education among old female populations. Indeed, the influence of partner characteristics on health has been shown to be significant in most European countries, although with differences in the magnitude of the importance of this effect, with countries in the south of the continent presenting the highest correlation (Banks et al., 2021).

To the best of our knowledge, this study is the first to analyze health transitions of old Europeans belonging to three birth cohorts, divided in three educational groups, and broken down by gender across four country groups by implementing a multistate approach. This technique has been used in panel data previously (Reuser et al., 2009); however, there are few precedents applied to European data. One obvious exception is the work of Reus-Pons and colleagues (2018), who analyze differences in health transitions between older European migrants and non-migrants. The approach taken here, however, not only contributes up-to-date evidence on the health transitions of older individuals across different European country groups, it also successfully incorporates an examination of the effects of birth cohort, educational attainment and gender differences on health transitions. The results of the multistate model concerning self-perceived health are reinforced when using GALI and ADL as health outcomes (Tables S1 to S4). These more objective health measures reaffirm the educational, age, gender and country group gradients found when using our subjective health outcome, but present different magnitudes in the probability transitions. In the three additional health outcomes considered, education influences the speed of transition within the disablement process that ends in death. This educational effect presents a similar pattern according to gender, birth cohort and country group. The importance of exploring further the mechanisms behind these transitions (particularly from good to poor health, and to death) is stressed by the significantly low probabilities of transitioning to death from a state of good health, regardless of the health measure used. 

Overall, our results highlight diverging patterns in the course of the disablement process. Thus, we find different speeds of aging for individuals with different characteristics. For instance, Northern and Central Europeans present a slower rate of transition between the different health states (including the recovery of good health), while Europeans from the east present faster rates of transition towards health deterioration and, finally, death. We detect that education exerts a significant effect, particularly, in Northern and Central Europe, by postponing transitions from good to poor health, while this effect is less evident for Eastern Europeans. Our findings not only reaffirm the different mortality patterns recorded across Europe (Movsisyan et al., 2020), but, more notably, they also reveal that morbidity patterns are key drivers in the acceleration or slowing down of the aging process before death. To explain the longevity of our society (not only its length but also its quality), we need to understand how these differences in the health process prior to death deteriorate among different demographic and socioeconomic groups.

Despite these strengths, the study is not without certain limitations. One potential limitation is the source of data we employed. As described above, the SHARE survey was first launched in 2004 and progressively added new countries and expanded the sample size across all participating countries. This means that we have been unable to employ the same time reference when analyzing our data, which prevents us from considering contextual information (e.g. 2008–2012 economic recession). Closely linked to this limitation is the fact that the incorporation of new countries, or their absence from certain waves, prevents us from analyzing the level of heterogeneity in terms of the health transitions analyzed within each country group. In addition, the fact that the SHARE survey does not include the institutionalized older population implies that our results might underestimate the real magnitude of some probabilities, especially in the case of the transition between poor health and death. Indeed, the latter has been identified as one of the most likely causes of underestimation of the magnitude of mortality when using the SHARE survey (Schulz & Doblhammer, 2011).

Furthermore, SHARE survey is not free of attrition, a common limitation in panel surveys. However, the profile of SHARE dropouts has shown a high level of heterogeneity without a defined pattern (Beller et al., 2022). Indeed, attrition has not been shown to alter the composition of the final sample with respect to the real one in the different countries that participate in the SHARE Survey (Friedel & Birkenbach, 2020). Finally, potential interaction effects between the proposed determinants of health and country have not been tested due to restrictions in the sample size once the analyses are stratified according to birth cohort, gender and country group. Conducting this analysis would have improved understanding of the significant effects of gender and education in the country groups considered. Despite this limitation, our transition to death results are consistent with the general patterns previously found for this indicator of population health (i.e. higher mortality values among men, a clear educational gradient, and higher overall mortality in Eastern European countries). Finally, this paper uses education as a factor to help understand the educational gradient in health and mortality. All factors considered in this study account for the most important contextual features related to health; however, we are aware of the importance of other individual characteristics factors, such as health-related behaviors or healthy lifestyles and attitudes, that are closely linked to educational status and health-related protective factors (Arcaya et al., 2015). Work is currently underway to analyze these factors as part of future studies.

Given the nature of our data, a selection effect in our outcomes cannot be totally discarded. Previous research based on SHARE data has shown that causation mechanisms prevail with respect to selection in the pathways between socioeconomic status and health as populations grow older (Hoffmann et al., 2018). Based on our health transition results, our future research plans include three additional approaches. First, we seek to analyze how intra group variation increases or decreases in relation to educational attainment. Second, we wish to estimate total and healthy life expectancy by population subgroups based on the results from the multistate life table method as this should shed light on different aging processes in the European country groups and help define more specific policies for different types of aging society. These findings moreover should be easily understood by policymakers and serve to address key public health policies. And third, we plan to demonstrate different gender equity levels in each country or group of countries as a possible modifiable factor of the different health transitions analyzed.

To conclude, education, gender, and country-group differences have a differential impact on the slowing down of the aging process, depending on the particular transition (i.e. deterioration, recovery, and transitions from either good or poor health to the absorbing state). This highlights the need to continue exploring the specific influence that individual factors have on health according to the individual and geographical context. Future public health policies need to prioritize actions that can postpone health deterioration and, in this way, improve the health and well-being outcomes of the older population.

## Supplementary Information

 Below is the link to the electronic supplementary material.

ESM 1 (DOCX 66.9 KB)

## Footnotes

1 http://www.uis.unesco.org/education/pages/international-standard-classification-of-education.aspx
