# Supplementary material for: (Healthy) Aging Patterns in Europe: A Multistate Health Transition Approach
Source: J Popul Ageing. 2022 Nov 25;16(1):179–201. doi: 10.1007/s12062-022-09403-4 (PMC9702679; doi:10.1007/s12062-022-09403-4)
Supplement: Supplementary file 1 — (DOCX 66.9 KB) [file 12062_2022_9403_MOESM1_ESM.docx]

**Table S1**: Activities of Daily Living (ADLs) transitions by country groups, year of birth and education level. Women aged 50 to 84

Source: Pooled data from the first to the seventh waves of SHARE (2004–2017).

**Table S2**: Activities of Daily Living (ADLs) transitions by country groups, year of birth and education level. Men aged 50 to 79

Source: Pooled data from the first to the seventh waves of SHARE (2004–2017).

**Table S3**: GALI transitions by country groups, year of birth and education level. Women aged 50 to 84

Source: Pooled data from the first to the seventh waves of SHARE (2004–2017).

**Table S4**: GALI transitions by country groups+, year of birth and education level. Men aged 50 to 79

**Table S5**: Health transitions (joint model). Women

Source: Pooled data from the first to the seventh waves of SHARE (2004–2017).

**Table S6**: Health transitions (joint model). Men

Source: Pooled data from the first to the seventh waves of SHARE (2004-2017).
